# Specific antibody titer decay in neonates prenatally exposed to *Toxoplasma gondii* and their mothers

**DOI:** 10.1186/s13052-022-01261-2

**Published:** 2022-05-03

**Authors:** Serena Salomè, Claudia Grieco, Pasquale Fabio Barra, Eleonora Capone, Fiorentino Grasso, Francesca Carraturo, Pasquale Dolce, Paola Salvatore, Letizia Capasso, Francesco Raimondi

**Affiliations:** 1 Division of Neonatology, Department of Translational Medical Sciences, University Federico II, Via Pansini 5, 80131 Naples, Italy; 2 Department of Public Health, University Federico II, Naples, Italy; 3 Department of Molecular Medicine and Medical Biotechnology, University Federico II, Naples, Italy

**Keywords:** Congenital toxoplasmosis, Antibody titer, Follow-up

## Abstract

**Background:**

For infants exposed in utero to *Toxoplasma gondii*, current guidelines recommend monitoring the specific antibody titer until 12 months of age. In this study, we investigated the antibody titer decay in the mother-infant dyad.

**Methods:**

This is a single center, population-based cohort study of neonates referred for prenatal exposure to *Toxoplasma gondii* from January 2014 to December 2020. All infants underwent clinical, laboratory, and instrumental investigation for at least 12 months.

**Results:**

A total of 670 eligible neonates were referred to the Perinatal Infection Unit of the University Federico II of Naples. 636 (95%) completed the serological follow up until 12 months. Specific IgG antibodies negativization occurred in 628 (98.7%) within 5 months. At 9 and 12 months, all patients had negative IgG. An initial neonatal IgG antibody titer ≥ 200 IU/ml was associated with a longer time to negativization (184 [177.5;256] days when above threshold vs. 139.5 [101;179] days when below it; *p* < 0.001). Maternal IgG antibody titer ≥ 200 IU/ml at childbirth was also associated to delayed time to negativization in the infant (179 [163;184] days above the threshold vs 125 [96.8;178] days below it; *p* < 0.001). Specific antibody negativization was irreversible in all patients.

**Conclusions:**

Lower anti-*Toxoplasma* antibody titers detected at birth in the mother-infant-dyad lead to an earlier and irreversible negativization. This information allows for customisation of the infant follow up program and avoids invasive and expensive tests.

## Background

Congenital Toxoplasmosis (CT) is an important cause of chorioretinitis, intracranial calcification, hydrocephalus, and potentially severe and permanent neurological deficits (psychomotor delay or other neurological deficiencies, seizures, and mental retardation) [1], although at birth most infants appear asymptomatic.

Soon after birth, infection is investigated clinically and instrumentally. The diagnosis mainly relies on positive IgM and/or IgA titers, detection of Toxoplasma gondii DNA in the amniotic fluid or cerebrospinal fluid (CSF), or positive IgM/IgA in CSF [2, 3]. When diagnostic criteria are not present at birth, the persistence of specific antibodies beyond 12 months of age is as an accepted demonstration of congenital infection [2, 3]. For this purpose, asymptomatic infants undergo periodic blood drawings that are both invasive and costly. A shorter, customized laboratory follow up would be desirable but reliable predictors are currently lacking.

The present population-based, cohort study investigates the hypothesis that initial lower anti-Toxoplasma antibody titers detected in the mother-infant-dyad may lead to an earlier and irreversible negativization. This information would provide a solid base for reducing pain towards infants and costs to the health system.

## Methods

Maternal infection was defined according to the Lebech’s classification system and case definition [4]. Seroconversion in pregnancy is coded as “1.1.1” case and presents a higher risk of vertical transmission. In selected cases, the diagnosis was confirmed by detecting Toxoplasma DNA using PCR technique on amniotic fluid. The extraction of *Toxoplasma gondii* DNA was performed with the Extrablood kit (ELITech group, Molecular Diagnostic s.p.a.) according to manufacturer’s instructions as previously reported [5]. The identification was carried out by Real Time PCR with the Toxoplasma g. Elite MGB kit (ELITech group, Molecular Diagnostic s.p.a.), which specifically detects a RE region of *Toxoplasma gondii* (a short sequence repeated 200 to 300 times in the microorganism genome).

Expectant mothers were then referred for a clinical and ultrasound follow up while on an appropriate treatment (either spiramycin or combination therapy with pyrimethamine, sulfadiazine, and folinic acid), according to specific guidelines [3]. Infants underwent an instrumental assessment of the central nervous system alongside an eye exam and auditory testing within the first month of life. Cranial Ultrasonography (US) was performed by an experienced neonatologist, who was blinded to the clinical data, using the Philips HD11 ultrasound imaging platform with 8.5–12.4 MHz transducers (Microconvex and Phased Array transducers). The fundus oculi examination was performed by a pediatric ophthalmologist skilled in congenital infections. Hearing function was evaluated by transitory evoked otoacoustic emissions (TEOAE) at birth and by automatic auditory brainstem response (ABR) after three months of life if the previous evaluation was not normal.

Paired serum samples from the mother and the neonate were collected for determination of IgG, IgA and IgM antibodies levels. The anti-*Toxoplasma* antibodies IgG and IgM, and IgG-Avidity in patients’ serum, were measured with an automated quantitative test using the VIDAS Instrument (Biomerieux SA). The assay principle combines a two-step enzyme immunoassay sandwich method with final fluorescent detection (Enzyme Linked Fluorescent Assay—ELFA). For the qualitative detection of anti-*Toxoplasma* IgA, the *in vitro* diagnostic kit PLATELIA™ TOXO IgA TMB (Bio-Rad) was used, it is a solid-phase immunoenzymatic double sandwich method, with capture of the IgA anti-*Toxoplasma* in the solid phase.

The results of IgG and IgM antibodies were expressed in International Units per ml (IU/ml) and were considered as negative when less than 8 IU/mL and 0.65 IU/mL, respectively. The IgG upper detection limit was 300 IU/mL. A titer equal to or greater than 300 IU/mL is generally considered high [4]. The results of the IgA antibodies were expressed by calculating the Simple Ratio according to the formula: Sample O.D._450/620 nm_ / mean O.D._450/620 nm_ Cut-Off. The sample was positive for Sample index ≥ 1.

Data was recorded on a standardized database.

### Patients

This is a prospective collection of data gathered from January 2014 to December 2020 at the Perinatal Infection Unit of the University “Federico II” of Naples, a tertiary care hospital with a dedicated multidisciplinary team.

Study inclusion criterion was the presence of positive neonatal IgG and negative IgM and/or IgA serology. Antibody determination was scheduled at 2 and 3 months of age and then every two months until complete negativization. The latter was confirmed after 30 days and a final antibody determination was provided at one year of life [3]. A different schedule was followed in case of a non-reassuring titer decrease.

Exclusion criteria included: parental denial to participate; neonates with history of detection of *Toxoplasma gondii* DNA in amniotic fluid and/or a positive anti-*Toxoplasma* IgM and/or IgA serology who then received an early diagnosis of active CT [2, 3]; evidence of CT after complete clinical, radiologic, and laboratory evaluation; infants who did not complete the 12 months follow up.

### Data analysis

Statistical analysis was performed using the R software environment for statistical computing.

Data is presented as a Median (I Quartile; III Quartile) for quantitative variables and as frequency (percentage) for categorical variables.

For categorical variables, the test for significant associations was performed using the chi-square test with continuity correction. Correlation between quantitative variables was analyzed using the Pearson correlation coefficient. Comparisons between groups for quantitative variables were performed using the Wilcoxon Test and Bonferroni method for multiple comparison correction.

*P* value less than 0.05 was considered as statistical significance.

## Results

### Population characteristics (Fig. 1)

**Fig. 1:**
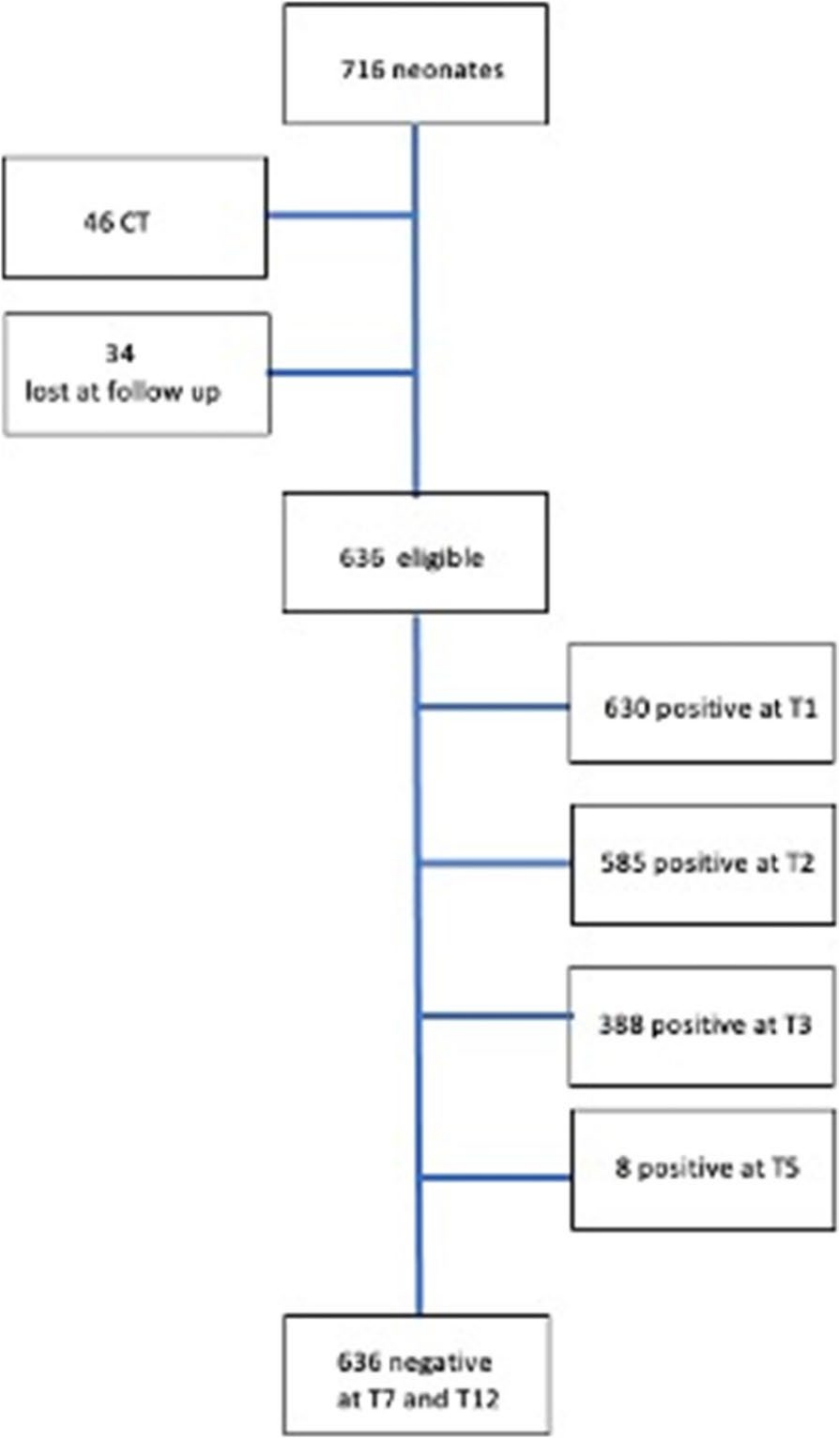
Population in study. This figure shows the population enrolled in the study

During the study period, 46 diagnoses of CT were made at the Perinatal Infection Unit through prenatal diagnosis (positive amniocentesis), positive IgM and/or IgA at birth, or specific clinical signs and therefore were not included in the study. Out of a total of 670 neonates responding to inclusion criteria, 636 (95%) completed the follow up until 12 months and were considered for the study.

Data on maternal history and neonatal characteristics are summarized in Table 1. In most cases infection occurred in the first trimester (467 cases, that is 73.4%). In 79 cases (12.4%) a seroconversion during pregnancy was demonstrated (Lebech’s classification “1.1.1”): 21 in the first trimester, 37 in second trimester, and 21 in the third trimester. Unsafe eating habits were reported in 119 mothers (19%); 52 women (8%) were pet owners and had higher IgG titers at childbirth compared to those who did not have pets (137 [78;205.8] vs 101 [63.8–164.2], *p* = 0.027). Maternal IgG titers at childbirth were not statistically associated with presence of symptoms in pregnancy or unsafe heating habits.

**Table 1:** Maternal and neonatal characteristics. This table shows the main characteristics of mother and children enrolled in the study

Lebech’s classification of maternal infection	
111	79 (12.4%)
Others	541 (85.1%)
Not known	16 (2.5%)
Maternal country of birth	
Italy	591 (92.9%)
European Union	36 (5.7%)
Others	9 (1.4%)
Maternal age (years)	30.1 ± 5.3 (15–45)
Specific symptoms in pregnancy	
Yes	63 (9.9%)
No	408 (64.2%)
Not known	165 (25.9%)
Prenatal ultrasound scan	
Normal	630 (99.1%)
Pathological	6 (0.9%)
Amniocentesis	
Negative for Toxoplasma DNA	151 (23.7%)
Positive for Toxoplasma DNA	0
Not performed	485 (76.3%)
Specific prenatal therapy	
Yes	505 (79.4%)
No	131 (20.6%)
Time of maternal infection	
Periconceptional period	28 (4.4%)
First trimester	467 (73.4%)
Second trimester	78 (12.3%)
Third trimester	41 (6.4%)
Undetermined	22 (3.5%)
Neonatal gender	
Male	326 (51%)
Female	310 (49%)
Neonatal gestational age at birth (weeks)	38.5 ± 1.6 (29–42)
Neonatal birth weight (grams)	3127 ± 489.3 (1310–4840)

### Management and follow-up

The first blood sample collected (T0) showed an IgG titer higher than 300 IU/mL in 35 mothers (5.5%) and 10 of their neonates (1.6%). Among these 35 mothers, 5 (14.3%) were classified as “1.1.1” according to Lebech; none of the 10 neonates with an IgG titer higher than 300 IU/mL was born to a mother with a seroconversion in pregnancy. Both higher maternal and neonatal IgG titers were not related with a high-risk class according Lebech’s classification (p 0.595 and p 0.410 respectively). After one month (T1) and two months (T2) from birth, specific IgG were positive in 630 (99.1%) and 585 cases (92%), respectively. At three months (T3) 388 neonates (61%) had still specific IgG. At 5 months (T5) only 8 (1.3%) were still positive. At 7 (T7) and 12 (T12) months all neonates were negative (Fig. 1). Time to negativization was not related to a high-risk class according Lebech’s classification (*p* = 0.108).

Considering the small number of mothers and neonates with an antibody concentration over 300 UI/mL, we used a threshold of 200 UI/mL for our subsequent analysis.

**Fig. 2:**
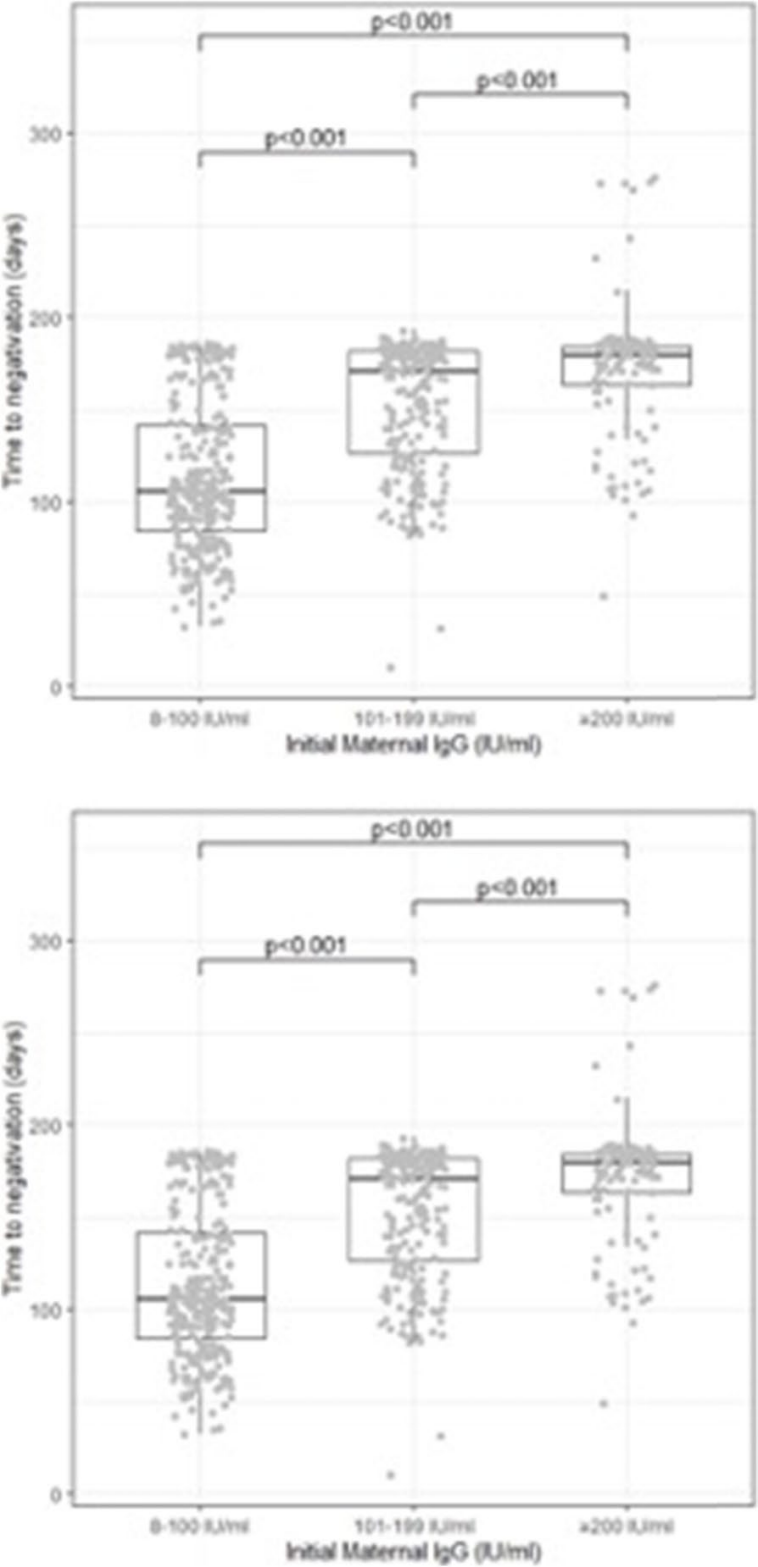
Time to negativization for intervals of maternal and neonatal IgG titer. This figure shows time to negativization of maternal and neonatal IgG titer according different levels of IgG

The neonatal initial titer was related to maternal antibody concentration at birth (*r* = 0.71; *p* < 0.001). Time to negativization was directly related to maternal and neonatal initial IgG value (*r* = 0.71, *p* < 0.001 and *r* = 0.60, *p* < 0.001, respectively). Moreover, time to negativization was directly related to maternal titer at delivery and initial neonatal titer, categorizing the population of mothers and child in three groups based on intervals of IgG (8–100 UI/mL, 101–199 UI/mL, ≥ 200 IU/mL), as shown in Fig. 2. Considering maternal titer at delivery, time to negativization in child has a median of 105 days [84;141] in the first group, 171 days [125;182] in the second, and 179 days [163;184] in the third. Considering neonatal initial titer, time to negativization in child has a median of 125 days [98;178] in the first group, 179 days [172;184] in the second and 184 days [177.5;256] in the third.

Positivity at 180 days of life was statistically associated to maternal antibody value at delivery above the threshold of 200 UI/mL (12.6% vs 35.6%, *p* < 0.001). It was also statistically significant if we considered the neonatal value at birth (1.2% vs 9.1%, *p* < 0.001).

In particular, the 518 babies whose mothers had an IgG titer at delivery < 200 UI/mL negativized specific IgG earlier than the 118 babies whose mothers had with an IgG titer ≥ 200 UI/mL [125 days (96.8; 178) vs 179 days (163; 184) – *p* < 0.001]. Moreover, the 615 babies with an IgG titer < 200 UI/mL at first check negativized specific IgG earlier than the 21 babies with an IgG titer ≥ 200 UI/mL [139.5 days (101; 179) vs 184 days (177.5; 256), *p* < 0.001].

Negativization of specific IgG was irreversible in all tested babies.

## Discussion

CT can be diagnosed at birth in presence of a definite serological status and clinical manifestations. The overall incidence of seroconversion and suspected infection in pregnancy is 0.8 per 1,000 live births and the incidence of congenital toxoplasmosis 0.1 per 1,000 live births when applying a strict protocol of screening, follow-up, and treatment in pregnant women [6]. Assuming an overall mother to infant transmission rate of 25% [2], up to 75% of infants without congenital infection present with a positive serology at birth. Based on expert consensus, a congenital infection is categorically excluded if specific IgG are negative at twelve months of life. In order not to miss any late diagnosis and subsequent delayed treatment with negative effect on prognosis, these infants undergo a minimum of five laboratory evaluations [2, 3].

On a large cohort, we show a significant correlation between maternal and neonatal initial IgG titer and the infant’s age at antibody negativization suggesting that in children with rapidly undetectable antibodies, it could be considered to reduce the number of laboratory evaluations. In our population at 5 months only 8 infants (1.3%) were still positive and by 7 months all were negative. All infants with a later negativization had mothers with an IgG titer at delivery of ≥ 200 UI/mL and themselves had an IgG titer ≥ 200 UI/mL initially. Moreover, no child showed a recurrence of previously negative specific IgG.

Even in case of late maternal seroconversion, that is a condition at higher risk of infection for the fetus, a low maternal and neonatal titer at birth was related to early negativization in the infant without later increasing of IgG and subsequent diagnosis of CT.

Maternal seroconversion in the third trimester is a known condition at higher risk of infection for the fetus, in our series, 17 out of 21 of such late seroconversions had a low (i.e., < 200UI/ml) antibody titer. Interestingly, no infant was diagnosed with CT as they all showed an early negativization without anti Toxoplasma IgG rebound.

Based on these data, whenever negativization occurred, it would be suggestable to repeat serological evaluation directly at one year of life thereby reducing strain on the patient and their family, and the health care system. We calculate that, given a cost of 20 euros per laboratory work up, the total expenditure sums up to 140 euros per patient. Reducing serological evaluation for an average number of 150 patients per year, could potentially save 5,500 euros annually.

Our data suggest that a slightly simplified lab work schedule can be recommended once negativization occurs. This implies a small, yet appreciable reduction in patient stress and hospital costs.

We recommend the current follow up protocol for dyads with initial antibody titers above the 300 IU/ml threshold.

An implemented protocol for an early CT diagnosis may also be achieved with more specific blood tests. Capobiango JD et al. [7] have described, an immunoblot test for the comparison of mother and infant IgG and IgM immunological profiles at birth or shortly after. Mahmoudi S et al. characterized an interferon-gamma release assay (IGRA) where an early CT diagnosis was achieved by stimulating T-cells from infected neonates with *T. gondii* antigens [8]**.**

The latter test has shown promising results in our preliminary experience that is currently limited due to the high costs of the laboratory procedure.

## Conclusions

In conclusion, babies prenatally exposed to *T. gondii* with a specific titer at birth below 200 IU/ml may safely benefit of a follow up program consisting in serological evaluation till negativization and then directly at twelve months of life, thus significantly sparing infants from stress and valuable savings for the health care system.

## Data Availability

The dataset generated and analyzed during the current study is not publicly available because individual privacy could be compromised but are available from the corresponding author on reasonable request.

## Abbreviations

CT: Congenital Toxoplasmosis
CSF: Cerebrospinal fluid
US: Cranial Ultrasonography
TEOAE: Transitory evoked otoacoustic emissions
ABR: Automatic auditory brainstem response
ELFA: Enzyme Linked Fluorescent Assay
IGRA: Interferon-gamma release assay
